# Elevational Patterns of Species Richness, Range and Body Size for Spiny Frogs

**DOI:** 10.1371/journal.pone.0019817

**Published:** 2011-05-17

**Authors:** Junhua Hu, Feng Xie, Cheng Li, Jianping Jiang

**Affiliations:** Chengdu Institute of Biology, Chinese Academy of Sciences, Chengdu, Sichuan, China; Texas A&M University, United States of America

## Abstract

Quantifying spatial patterns of species richness is a core problem in biodiversity theory. Spiny frogs of the subfamily Painae (Anura: Dicroglossidae) are widespread, but endemic to Asia. Using spiny frog distribution and body size data, and a digital elevation model data set we explored altitudinal patterns of spiny frog richness and quantified the effect of area on the richness pattern over a large altitudinal gradient from 0–5000 m a.s.l. We also tested two hypotheses: (i) the Rapoport's altitudinal effect is valid for the Painae, and (ii) Bergmann's clines are present in spiny frogs. The species richness of Painae across four different altitudinal band widths (100 m, 200 m, 300 m and 400 m) all showed hump-shaped patterns along altitudinal gradient. The altitudinal changes in species richness of the Paini and Quasipaini tribes further confirmed this finding, while the peak of Quasipaini species richness occurred at lower elevations than the maxima of Paini. The area did not explain a significant amount of variation in total, nor Paini species richness, but it did explain variation in Quasipaini. Five distinct groups across altitudinal gradient were found. Species altitudinal ranges did not expand with an increase in the midpoints of altitudinal ranges. A significant negative correlation between body size and elevation was exhibited. Our findings demonstrate that Rapoport's altitudinal rule is not a compulsory attribute of spiny frogs and also suggest that Bergmann's rule is not generally applicable to amphibians. The study highlights a need to explore the underlying mechanisms of species richness patterns, particularly for amphibians in macroecology.

## Introduction

The diversity of animal and plant species on Earth is not uniformly distributed along latitudinal and altitudinal gradients [1], and geographical gradients of diversity have long fascinated biogeographers and ecologists [2], [3]. Altitudinal gradients yield consistent ecological conditions and histories and are linked to several environmental variables of interest to theoretical and applied research on biodiversity. In particular, focus has been placed on altitudinal gradients [4]. Along altitudinal gradients, the species richness-altitude relationship generally follows a decreasing or hump-shaped pattern, depending on the main attributes of scale (i.e. the unit of sampling and the geographical space covered) [5]. However, growing evidence suggests that the uniformly decreasing pattern is less common than the hump-shaped pattern [5]–[8]. Understanding altitudinal pattern in species richness offers a fascinating opportunity to investigate the general mechanisms responsible for the distribution of biodiversity [5], [9], [10].

Climatic, biological, geographical and historical factors impact upon observed species richness-altitude patterns [6], [7], [11], [12]. The altitudinal gradient of species richness may be intricately related to species-area relationships [7], [13]. The effect of area on species richness has been described as one of ecology's few laws [14] and under the area hypothesis larger regions are expected to be more diverse than smaller regions [11], [15]. The species-area relationship can be accounted for by two principle hypotheses: (1) a greater area provides greater habitat diversity which can harbor more species [16], and (2) increases in area are accompanied by decreased rates of extinction and increased rates of speciation or colonization due to a greater number of barriers and the maintenance of larger population sizes [15], [17]. Typically, the hypothesis asserted varies with the spatial size, where habitat diversity is often considered the primary driver at local to landscape scales and the processes of colonization and extinction predominate at larger regional to global scales [11]. It is suggested that the area of altitudinal belts explain a large proportion of the variation in species richness [18]–[21].

Rapoport's rule states that there is a positive relationship between the latitudinal/altitudinal geographical range of an organism and latitude/altitude [22], [23]. ‘Rapoport's altitudinal rule’ was explained in terms of the differential ability of species to attain large range sizes. Species at low elevations are approaching their upper elevation range limits, while species that inhabit higher elevations have comparatively larger climatic tolerances and thus can be found across a greater altitudinal range [22]. Unfortunately, conclusions on the generality of Rapoport's rule are precluded by the uneven taxonomic and latitudinal representation of organisms examined thus far [24]–[28].

The tendency for organisms in cooler climates to be larger in size (Bergmann's rule) is well-documented for endotherms (birds and mammals) [29]–[31], and is reputed to apply to some ectotherms, including amphibians (e.g., some salamanders, newts and anurans) [32]–[34]. However, the general applicability of this rule (to both ectotherms and endotherms) has been vigorously debated as evidence exists for both Bergmann and converse Bergmann clines. There is also evidence of inconsistent biogeographical patterns in various groups of ectotherms including fishes, amphibians and reptiles [32], [35]–[38]. While it was questioned whether Bergmann's clines are present in amphibians [39], they are particularly interesting for evaluating the generality of geographical patterns of body size variation, and understanding underlying mechanisms [32], [34], [40]. Adams and Church [39] suggest that resolving this question for amphibians is an important step in understanding the evolution of body size clines in vertebrates.

To address these issues we used spiny frogs of the subfamily Painae (Anura: Dicroglossidae) [41] as a case study and examined frog species diversity over a large altitudinal gradient. Despite a large number of studies on the phylogenetics, classification and historical biogeography of spiny frogs [41]–[45], large-scale distribution patterns are not well understood and many questions remain. For example, what are the patterns of species richness along altitudinal gradients? Are patterns consistent across different altitudinal bands? Are there Rapoport's altitudinal effects? Do spiny frogs follow Bergmann's rule? We explored the frog richness-altitude relationship, and also sought to assess the ability of area to explain altitudinal patterns of species richness and to test Rapoport's altitudinal rule and Bergmann's rule for spiny frogs. Through the collection of this important data, we hope to incite comprehensive research of ecological biogeography and to understand the general mechanisms responsible for the distribution of these model species and other amphibians.

## Methods

### Study taxa

Spiny frogs previously belong to the tribe Paini, which was first proposed by Dubois [46]. These frogs comprise a major group of amphibians and are endemic to Asia. The evolutionary tree of spiny frogs is well explored, and their classification has been well documented [41]–[44]. Forty-one species of spiny frogs, including some newly described, have been recognized. These frogs belong to the newly created subfamily Painae, which originated approximately 60 Ma [45] and branched into two tribes, Paini and Quasipaini, containing 33 and eight species respectively [41]. Spiny frogs live mostly in swift boulder-strewn streams in the mountains across the Himalayas and southern Qinghai-Tibet Plateau, Hengduan Mountains, northern Indochina, and southern and central China [47], [48]. Their current distribution appears to be closely related to specific tectonomorphological features, including the Qinghai-Tibetan Plateau, Himalayas, Hengduan Mountain Range, and Indochina [45] and include three biodiversity hotspots [49]. Given that the ecological gradients provided are broad, they are particularly interesting study sites and can serve as templates for mountainous regions worldwide. However, a comprehensive study on spiny frog diversity in relation to elevation is lacking, and only ancillary information is available: the distribution range along elevation is particularly wide, almost 5000 m a.s.l. [47], [48]. Because the Painae is monophyletic, widespread but endemic to a single land mass, and this group shows a great deal of variation in range size and susceptibility to changes in their environment [47], [48], spiny frogs represent an ideal clade for large-scale studies of diversity and distribution.

### Data sources

A database was generated from specimens collected by the Chengdu Institute of Biology, Chinese Academy of Sciences, our field surveys, and Muséum national d'Histoire naturelle of France (measured by Jianping Jiang in Paris under the care of Dubois and Ohler), and current literature [47], [48], [50]–[53]. Following the methods of Olalla-Tárraga and Rodríguez [34], we used maximum snout to vent length (SVL) as an estimate of body size. We compiled the body size and altitudinal distribution data (minimal and maximal elevation of occurrence) for each species.

**Figure 1:**
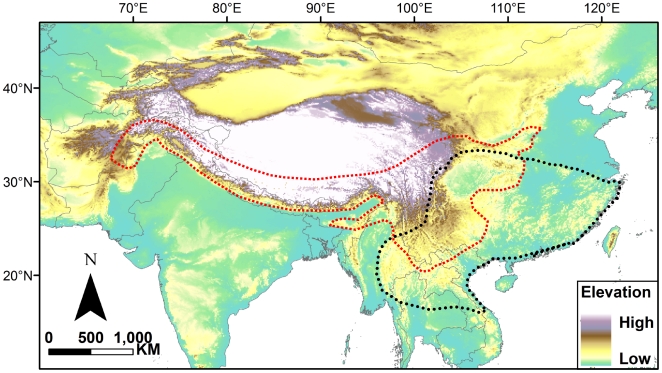
The sketch map of the study region in Asia. Current distribution ranges are indicated for the tribes Paini (red dotted line) and Quasipaini (black dotted line).

The area at a 200-m interval within the study region (Fig. 1) was calculated based on a global digital elevation model (DEM, GTOPO30) from the United States Geological Survey's Hydro1K dataset (http://edcdaac.usgs.gov/gtopo30/hydro/), with the resolution of a grid cell of 1×1 km. We extracted the map, which contained altitudinal information of the target regions, from the global GTOPO30 data. The area is a product of grid number by grid area.

### Statistical analysis

With an altitudinal range of 5000 m a.s.l., spiny frogs provide one of the broadest altitude gradients in the world for analyzing altitudinal patterns of species diversity. To examine the relationship between frog species richness and elevation, we divided the elevation range into different altitudinal gradients (100 m, 200 m, 300 m and 400 m band widths) and calculated the number of species in each band at different gradients. A species was assumed to have continuous ranges between its minimum and maximum altitudinal records.

We used the area data to examine the influence of area on the patterns of species richness along altitudinal gradient and to assess the relationship between species density (i.e. the number of species adjusted for area) and elevation, and between species richness and area. We calculated species density for altitudinal bands based on the following equation [54], [55]: 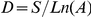, where *D* is species density, and *S* and *A* are the number of species and area in each altitudinal band, respectively.

To overcome statistical non-independence of the spatial data, we used the ‘mid-point method’ [56] as a measure of the central tendency. The mean between the minimum and maximum elevation reported for each species was used to represent that species' altitudinal range midpoint. Values of the range midpoint and breadth were used to examine relationships between the midpoints and breadths.

We compared community composition among elevation bands (200 m intervals) to explore the altitudinal pattern of community composition. The Jaccard (1901) index [57] was used to conduct the analysis of similarity measure. We computed pair-wise similarities among all bands to compose a similarity coefficient matrix and used the method of between-groups linkage in the cluster analysis based on this matrix.

To determine relationships between body size and elevation, we quantified the body size-elevation relationships for the Painae, Paini and Quasipaini. For all analyses, body size data were *log_10_* transformed and a length-frequency distribution was computed from these data.

Graphical analysis was used to explore patterns in species richness, altitudinal range and body size of spiny frogs. We used Kolmogorov-Smirnov tests to check for normality of data and we transformed the data to meet assumptions of normality. Parametric analyses were used to compare differences between data sets. We compared differences in body size between the two evolutional clades using the Independent-Samples T Test. Bivariate analyses were conducted and the Pearson correlation coefficient was used to express the sign and strength of the relationship between species richness and elevation or area, and between species density and area. The simple ordinary least squares (OLS) model was used to analyze associations between the considered parameters (range midpoint and breadth or body size). All analyses were done using SPSS 16.0 (SPSS, Chicago, USA). Data were presented as mean ± SE and *p*≤0.05 was considered statistically significant.

## Results

### Elevational patterns of species richness

Spiny frogs were distributed over a large altitudinal range with the highest altitudinal distribution of *Nanorana parkeri* up to 5000 m a.s.l. The most species-rich genus was *Paa*, with nine species. There were only three species above the forest-limit ecotone (above 4000 m a.s.l.) representing the genus *Nanorana*.

**Figure 2:**
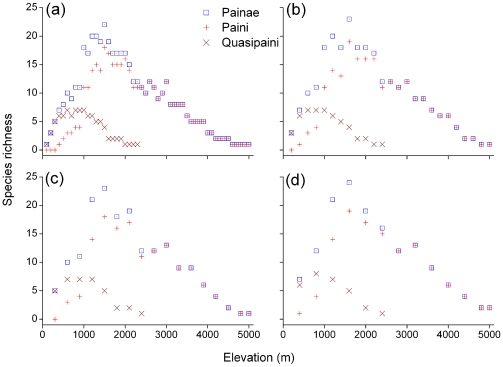
Elevational patterns of species richness of spiny frogs. Patterns are shown for the subfamily Painae (*n* = 41) and the tribes Paini (*n* = 33) and Quasipaini (*n* = 8) along the four altitudinal gradients: (a) 100 m interval, (b) 200 m interval, (c) 300 m interval and (d) 400 m interval.

Species richness for the subfamily Painae, the tribes Paini and Quasipaini showed a hump-shaped pattern along altitudinal gradient: richness increased steeply, and then decreased after peaking at intermediate elevations of their altitudinal ranges (Fig. 2a). Peaks in Quasipaini species richness occurred at lower elevations (600–1000 m a.s.l.) than the maxima of Painae or Paini species richness (both *c.* 1500 m a.s.l.). This humped pattern of species richness with elevation was consistent across all the four altitudinal band widths (Fig. 2a–d).

**Figure 3:**
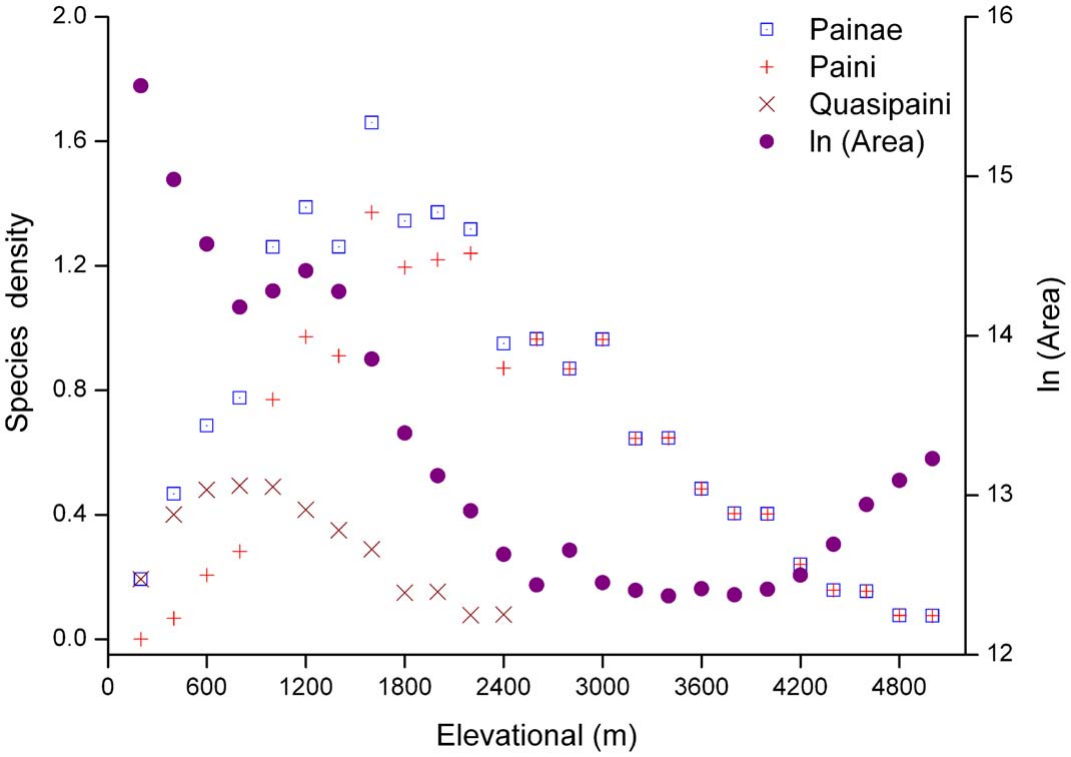
Changes in area and species density of spiny frogs along altitudinal gradient. Species density is the number of species per log-transformed and is shown for the subfamily Painae and the tribes Paini and Quasipaini respectively.

**Figure 4:**
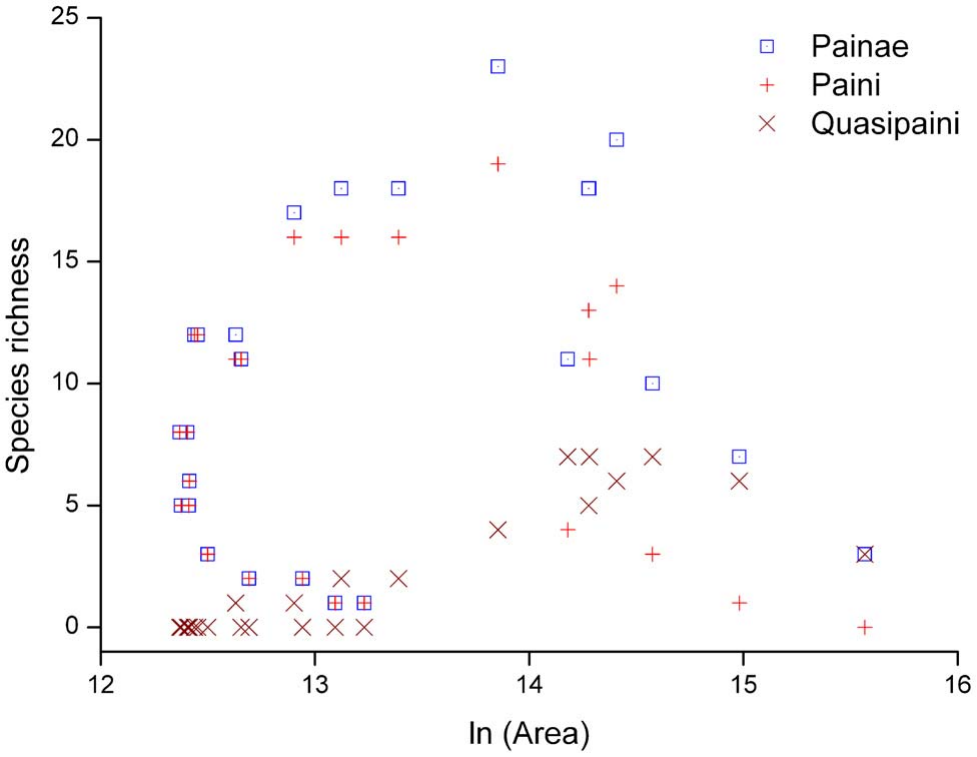
Scatter plots showing the relationship between species richness of spiny frogs and area. The relationship is shown for the subfamily Painae and the tribes Paini and Quasipaini respectively.

With increasing elevation, the area of each band decreased with fluctuations (*r* = −0.703, *p*<0.01; Fig. 3). The area of altitudinal bands decreased steeply from 0–800 m a.s.l, increased slightly in 800–1200 m a.s.l., and decreased after reaching a maximum at an elevation of 1200 m a.s.l. Finally, the area of each band above 4200 m a.s.l. gradually increased, possibly due to the existence of the Qinghai–Tibetan Plateau within the region. The correlation between species richness of total spiny or Paini frogs and area was not significant (both *p*>0.05), and maximum frog species richness did not occur below 600 m a.s.l., the range with the largest available area (Figs. 2, 3, and 4). Quasipaini frog richness was positively correlated with area (*r* = 0.598, *p*<0.01; Fig. 4).

Species density indicated similar altitudinal patterns to that of species richness for the Painae, Paini and Quasipaini frogs (Fig. 3). The species density peaks of Paini and Quasipaini frogs did not coincide. The maximum species density of Painae and Paini frogs both appeared around 1600 m a.s.l., while Quasipaini species density peaked between 800 m and 1000 m a.s.l.

**Figure 5:**
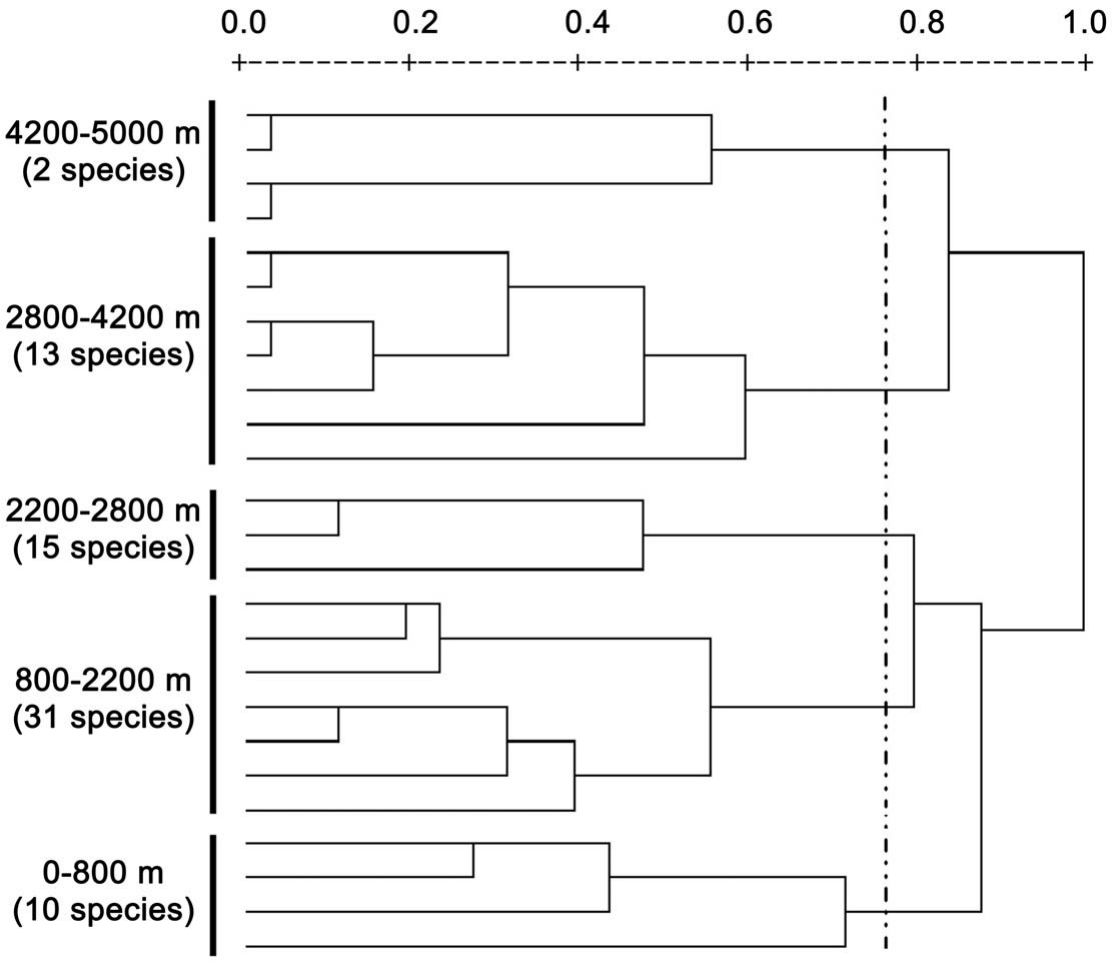
The classification of 200-m altitudinal intervals between 0 and 5000 m for spiny frogs. The Jaccard (1901) similarity measure is used. The between-groups method is used for the cluster analysis based on the similarity coefficient matrix.

Cluster analysis revealed five distinct groups along altitudinal gradient (Fig. 5). The altitudinal boundaries of the five groups were: (1) 0–800 m, (2) 800–2200 m, (3) 2200–2800 m, (4) 2800–4200 m and (5) 4200–5000 m. The number of species in 800–2200 m was much larger than in the other four groups.

### Elevational range size

**Figure 6:**
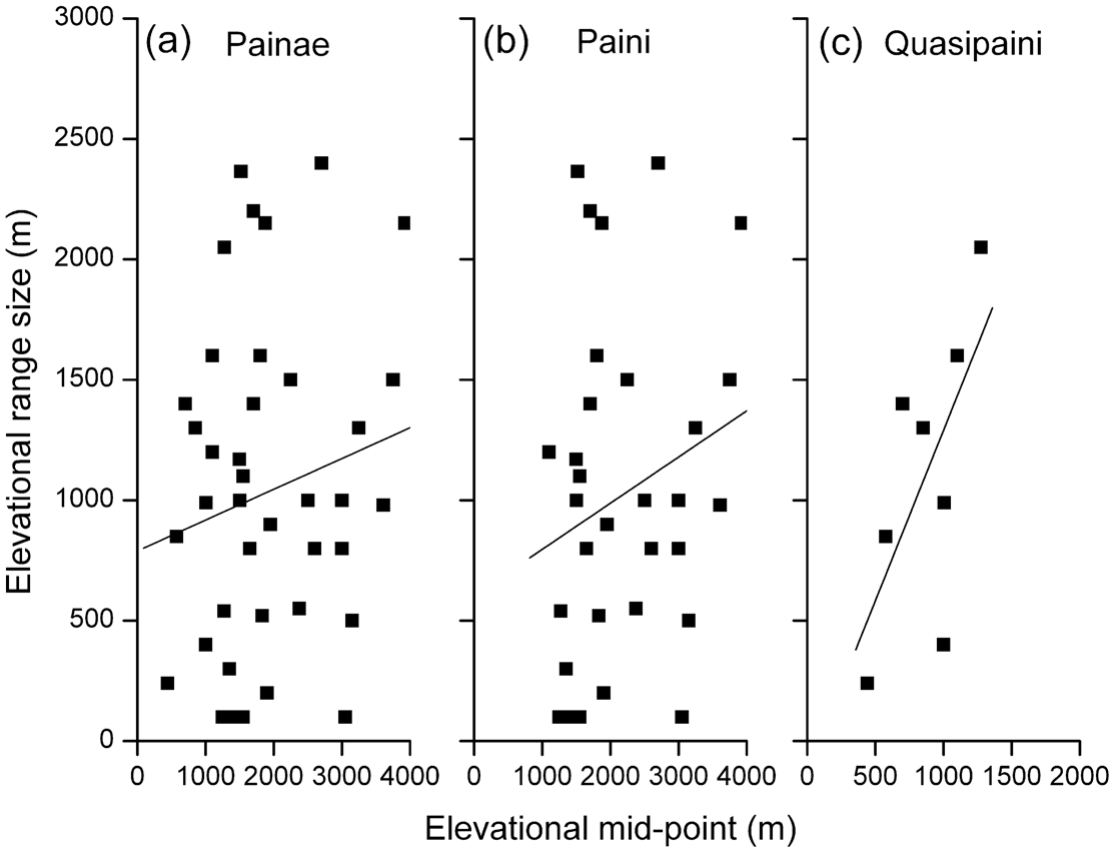
Relationship between altitudinal range midpoints and range size of spiny frogs. The relationship is shown for (a) the subfamily Painae, (b) the tribe Painiand and (c) the tribe Quasipaini respectively. The fitted line represents an ordinary least square (OLS) linear regression.

The altitudinal range of spiny frogs did not tend to increase with increasing elevation, rejecting Rapoport's rule (*n* = 41, *r* = 0.171, *p*>0.05; Fig. 6a–c). Even though there was less scatter around the best fit line for Quasipaini than for Paini, there was no positive correlation between the altitudinal range size and the range midpoint for the two tribes; species at higher elevations did not have broader ranges.

### Body size

**Figure 7:**
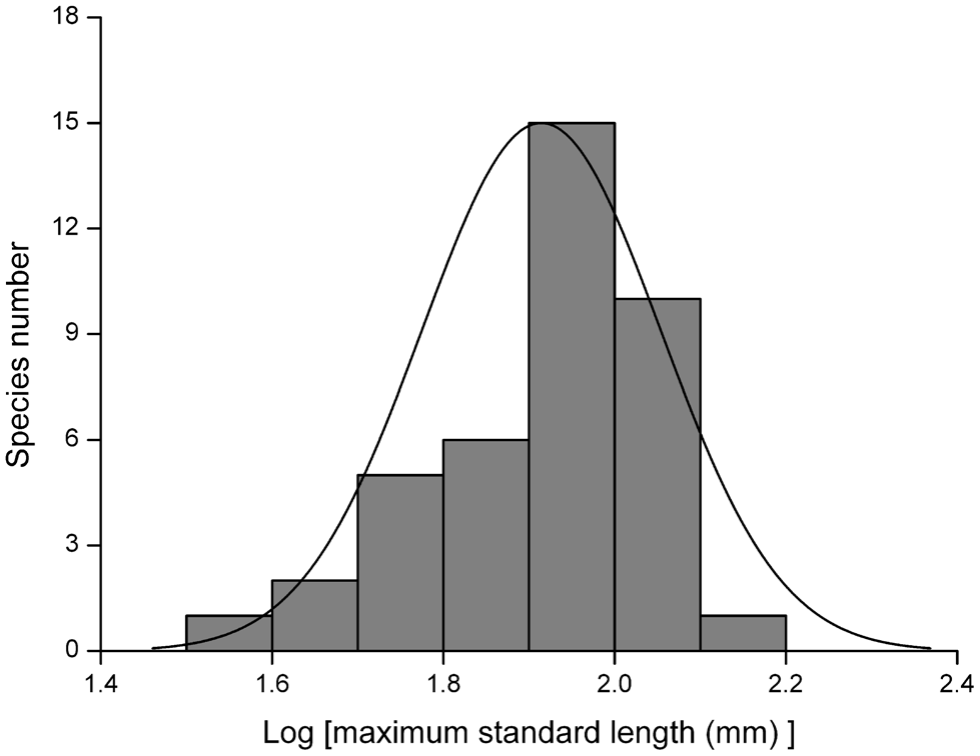
Frequency distribution of *log* maximum snout to vent length for spiny frogs (*n* = 41).

For total spiny frogs, the frequency distribution of *log* SVL data was normally distributed (Kolmogorov-Smirnov Z = 0.952, *p* = 0.325), and did not lose symmetry (Fig. 7). The curve was ‘smooth’ with more organisms possessing medium body sizes than adjacent body size categories.

**Figure 8:**
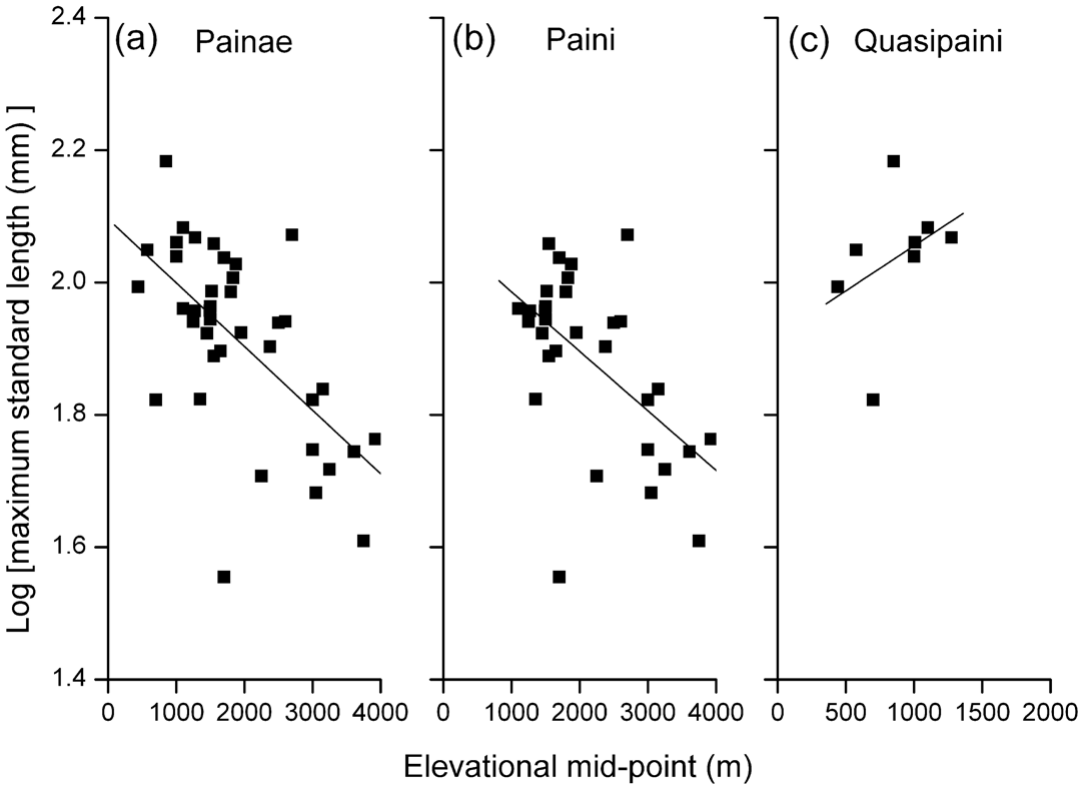
Relationship between altitudinal range midpoints and body size of spiny frogs. Maximum snout to vent length (SVL) is used as an estimate of body size. The relationship is shown for (a) the subfamily Painae, (b) the tribe Paini and (c) the tribe Quasipaini respectively. The fitted line represents an ordinary least square (OLS) linear regression.

Maximum SVL of spiny frogs varied significantly among species (86.03±3.99; *t* = 86.76, *p*<0.01). The greatest range of body sizes occurred at moderate elevations, and intermediate body sizes of *log* equal to approximately 2.0 occurred across the greatest range of elevations, while smaller and larger body sizes possessed only small altitudinal amplitudes. The SVL of Paini frogs (79.66±3.80) was smaller than that of Quasipaini frogs (111.51±8.46; *t* = −3.08, *p*<0.01). Correlation between the SVL of total spiny frogs and the altitudinal range midpoints was well explained by a simple ordinary least squares (OLS) model (*r* ^2^ = 0.389, *p*<0.01; Fig. 8a). An analogous association for the altitudinal range midpoints and the SVL of Paini was also well explained by an OLS model, with a slightly lower determination coefficient (*r* ^2^ = 0.318, *p*<0.01; Fig. 8b). There was no significant correlation between the altitudinal range midpoints and the SVL of Quasipaini (*r* = 0.374, *p*>0.05; Fig. 8c).

## Discussion

### Elevational patterns of species richness

It is crucial to understand species richness-altitude relationships for the development of a general theory on species diversity [4]. For herpetofauna, some studies suggest that species richness decreases monotonically with increasing elevation [58]–[60], while some indicate hump-shaped relationships between species richness and elevation [61]–[63]. In this study, species richness of spiny frogs showed a hump-shaped pattern across a broad altitudinal range. Such a pattern prevailed across the four different altitudinal bands (100 m, 200 m, 300 m and 400 m) and was identified by different evolutional clades. We suggest that this consistency across different altitudinal scales or evolutional clades further corroborates the goodness of fit of the hump shape. Pattern between species density and altitude further confirmed this trend. Other research from parts of our study region such as plant diversity in the Nepal Himalaya, the Indian Western Himalaya and the Gaoligong Mountains [19], [54], [64], [65], frogs, lizards and snakes in the Hengduan Mountains [61], [66], and small mammal diversity on Mount Qilian [67], also reported similar altitudinal richness patterns, showing a richness peak at mid-elevations across different taxa.

Reduced surface area and greater division of topography could lead to more isolated populations and hence higher speciation rates or endemism with increasing elevation [68], [69]. Here, species richness patterns along altitudinal gradients for Paini and Quasipaini were different, and the maxima in richness for Paini frogs peaked at higher elevations. Despite a similar extent of occurrence for Paini and Quasipaini frogs (Fig. 1), they differed in their number of species (33 versus eight species). This can be explained by the diversification history of spiny frogs: vicariance explains species formation among major lineages within Paini while dispersal plays an important role among Quasipaini [45]. Massive mountains and deeply carved valleys induced by the Indo-Asian collision and the continued uplift of the Himalayan region [70] exist in the distribution range of Paini and act as barriers to distribution, resulting in speciation and species diversification. However, the common ancestor of Quasipaini was likely distributed in present-day Indochina, and dispersed from Indochina to South China. The present distributional pattern of Quasipaini could be explained by clues from tectonic events, such as orogenic movement of the Truong Son Mountain Range and ductile movements of the Red River zone [45].

Species density of spiny frogs here showed two peaks at mid-elevations. This pattern was largely in accordance with the prediction of Lomolino [7]. He predicted that species density should peak at an intermediate elevation and the peak should occur at a transition zone between the two species-rich, juxtaposed communities. In addition to this major peak, species density should exhibit repeated minor peaks at transitions between other zonal communities. The cluster analysis of community similarity for spiny frogs revealed pronounced and distinct groups along altitudinal gradient [66], [71]. This indicates that the frog community composition pattern was strongly influenced by the basin stepped geomorphology. The cluster analysis based on community similarity also revealed a high altitudinal species group largely distinct from those of lower elevations for spiny frogs.

### Elevational range size

Species ranges result from complex interactions among many factors, including physiological traits, history of speciation and dispersal, and constraints from continent shape [72]. It remains unclear whether the general trends of Rapoport's rule exist for all biological organisms [73]. In this study, the altitudinal range size of spiny frogs did not increase with increasing elevation and this does not support Rapoport's altitudinal rule [22]. Distributions of Paini and Quasipaini frogs also showed similar patterns. Why does Rapoport's altitudinal rule not apply to spiny frogs? According to Stevens [22], Rapoport's altitudinal rule relates to the rescue effect and is presented as an explanation for monotonic decreases in species richness with increasing elevation. Spiny frogs showed peaks at intermediate elevations. It may be true that these apparently conflicting patterns cannot be reconciled.

However, Colwell and Hurtt [74] and Rahbek [6], [13] have pointed out that the data Stevens [22], [23] presented in support of his version of Rapoport's rule actually shows a peak at mid-elevations (or latitudes for the latitudinal version of the rule). The distribution of frogs reported here probably suffers from geometrical limits, and weakens the Rapoport effect by default [74]. Perhaps Rapoport's altitudinal rule describes a spurious effect, or if true, helps to explain peaks in species richness at mid-elevations rather than decreasing richness with increasing elevation. If anything, Rapoport's rule is not general [56], [75]. Our results complement other evidence indicating a lack of a Rapoport altitudinal pattern [13], [76], [77]. This suggests that the factors determining range size are complex and remain poorly understood [73].

### Area effect

Traditionally, the influence of area on species richness has been explained by the theory of island biogeography [15] or by the habitat diversity hypothesis [16]. However, these concepts are not mutually exclusive, and theoretically may even be complementary because area and habitat diversity are correlated [78]. Generally, in a region with larger area, habitat is more heterogeneous and diverse than in a region with smaller area, and thus can support more species. It has been widely observed that species richness increases as a function of area [13]. The influence of area in determining regional species richness in altitudinal ranges has been shown for different taxa [18], [20], [61]. For spiny frogs, the area of their distribution range showed a fluctuant pattern along the altitudinal gradient. This did not correspond to the altitudinal change in species richness. Furthermore, there was no significant correlation between species richness of Painae frogs and area along altitudinal gradient. The reasons for this may stem from the special relationship between area and elevation, greatly affected by the uplift of the Qinghai–Tibetan Plateau [70], [79].

### Body size

While Meiri and Dayan [80] suggest Bergmann's rule holds true for over 72% and 65% of bird and mammal species, respectively, inter-specific altitudinal variation in body size patterns that do not conform to Bergmann's rule have been reported in many studies [81]–[83]. For amphibians, empirical evidence supporting the prevalence of Bergmann's clines is still controversial [39]. Recent studies contest the claim that amphibians generally adhere to Bergmann's rule at the inter-specific level, some species exhibit body size clines consistent with Bergmann's rule, whereas other species lack the expected patterns [32]–[36], [39], [40], [84]. Ashton [32] showed that most amphibian species exhibited Bergmann clines with respect to latitude or altitude, although this trend was not significant within anurans. Olalla-Tárraga and Rodríguez [34] concluded that anurans follow a marked Bergmann's rule pattern and urodeles are the opposite. Here, the altitudinal body-size pattern of spiny frogs did not follow Bergmann's rule. This suggests that the question of whether Bergmann's clines should be generally present in amphibians remains unanswered [39] and more studies on body size-altitude relationships are needed before we can make generalizations on altitudinal variation in body size among amphibians.

Endemic species are of particular interest to conservation, management and biogeography [73]. While spiny frogs are widely distributed across Asia, the range of some species is restricted [47], [48] and their survival is threatened by habitat loss and illegal harvesting [85]. Moreover, evidence is accumulating that rapid climate change has already altered the distribution of many species [86], [87] and that further change is inevitable [88], [89]. High extinction rates around the world could occur [90]. To combat these problems, further studies into biogeography and management plans for spiny frogs are urgently needed.

## References

[1] Brehm G, Colwell RK, Kluge J (2007). The role of environment and mid-domain effect on moth species richness along a tropical elevational gradient.. Global Ecology and Biogeography.

[2] Lomolino M, Sax D, Brown J (2004). Foundations of Biogeography: Classic Papers with Commentaries.

[3] Hu J, Hu H, Jiang Z (2007). Distribution regularities of species diversity at large spatial scale.. Chinese Journal of Applied and Environmental Biology.

[4] Rowe R (2009). Environmental and geometric drivers of small mammal diversity along elevational gradients in Utah.. Ecography.

[5] Rahbek C (2005). The role of spatial scale and the perception of large-scale species-richness patterns.. Ecology Letters.

[6] Rahbek C (1995). The elevational gradient of species richness: a uniform pattern?. Ecography.

[7] Lomolino MV (2001). Elevation gradients of species-density: historical and prospective views.. Global Ecology and Biogeography.

[8] Grytnes J-A, McCain CM, Simon AL (2007). Elevational Trends in Biodiversity.. Encyclopedia of Biodiversity.

[9] McCain CM (2007). Area and mammalian elevational diversity.. Ecology.

[10] Beck J, Chey VK (2008). Explaining the elevational diversity pattern of geometrid moths from Borneo: a test of five hypotheses.. Journal of Biogeography.

[11] Rosenzweig M (1995). Species Diversity in Space and Time.

[12] Whittaker RJ, Willis KJ, Field R (2001). Scale and species richness: towards a general, hierarchical theory of species diversity.. Journal of Biogeography.

[13] Rahbek C (1997). The relationship among area, elevation, and regional species richness in neotropical birds.. American Naturalist.

[14] Lawton J (1999). Are there general laws in ecology?. Oikos.

[15] MacArthur R, Wilson E (2001). The Theory of Island Biogeography.

[16] Williams C (1964). Patterns in the Balance of Nature.

[17] Preston F (1962). The canonical distribution of commonness and rarity.. Ecology.

[18] Bachman S, Baker WJ, Brummitt N, Dransfield J, Moat J (2004). Elevational gradients, area and tropical island diversity: an example from the palms of New Guinea.. Ecography.

[19] Bhattarai KR, Vetaas OR, Grytnes JA (2004). Fern species richness along a central Himalayan elevational gradient, Nepal.. Journal of Biogeography.

[20] Kattan GH, Franco P (2004). Bird diversity along elevational gradients in the Andes of Colombia: area and mass effects.. Global Ecology and Biogeography.

[21] Körner C (2000). Why are there global gradients in species richness? mountains might hold the answer.. Trends in Ecology & Evolution.

[22] Stevens G (1992). The elevational gradient in altitudinal range: an extension of Rapoport's latitudinal rule to altitude.. American Naturalist.

[23] Stevens G (1989). The latitudinal gradient in geographical range: how so many species coexist in the tropics.. American Naturalist.

[24] Ribas CR, Schoereder JH (2006). Is the Rapoport effect widespread? Null models revisited.. Global Ecology and Biogeography.

[25] Gaston KJ, Chown SL (1999). Why Rapoport's rule does not generalise.. Oikos.

[26] Willig MR, Kaufman DM, Stevens RD (2003). Latitudinal gradients of biodiversity: pattern, process, scale, and synthesis.. Annual Review of Ecology, Evolution, and Systematics.

[27] Beketov MA (2009). The Rapoport effect is detected in a river system and is based on nested organization.. Global Ecology and Biogeography.

[28] Ruggiero A, Werenkraut V (2007). One-dimensional analyses of Rapoport's rule reviewed through meta-analysis.. Global Ecology and Biogeography.

[29] Ashton KG, Tracy MC, de Queiroz A (2000). Is Bergmann's rule valid for mammals?. American Naturalist.

[30] Ashton KG (2002). Patterns of within-species body size variation of birds: strong evidence for Bergmann's rule.. Global Ecology and Biogeography.

[31] Freckleton RP, Harvey PH, Pagel M (2003). Bergmann's rule and body size in mammals.. American Naturalist.

[32] Ashton KG (2002). Do amphibians follow Bergmann's rule?. Canadian Journal of Zoology.

[33] Krizmanic I, Vukov TD, Kalezic ML (2005). Bergmann's rule is size-related in European newts (*Triturus*).. Herpetological Journal.

[34] Olalla-Tárraga MÁ, Rodríguez MÁ (2007). Energy and interspecific body size patterns of amphibian faunas in Europe and North America: anurans follow Bergmann's rule, urodeles its converse.. Global Ecology and Biogeography.

[35] Lindsey CC (1966). Body sizes of poikilotherm vertebrates at different latitudes.. Evolution.

[36] Mousseau TA (1997). Ectotherms follow the converse to Bergmann's Rule.. Evolution.

[37] Ashton KG, Feldman CR (2003). Bergmann's rule in nonavian reptiles: Turtles follow it, lizards and snakes reverse it.. Evolution.

[38] Olalla-Tárraga MÁ, Rodríguez MÁ, Hawkins BA (2006). Broad-scale patterns of body size in squamate reptiles of Europe and North America.. Journal of Biogeography.

[39] Adams DC, Church JO (2008). Amphibians do not follow Bergmann's rule.. Evolution.

[40] Laugen A, Laurila A, Jonsson K, Soderman F, Merila J (2005). Do common frogs (*Rana temporaria*) follow Bergmann's rule?. Evolutionary Ecology Research.

[41] Fei L, Ye C, Jiang J (2010). Phylogenetic systematics of Ranidae.. Herpetologica Sinica.

[42] Jiang JP, Dubois A, Ohler A, Tillier A, Chen XH (2005). Phylogenetic relationships of the tribe Paini (Amphibia, Anura, Ranidae) based on partial sequences of mitochondrial 12s and 16s rRNA genes.. Zoological Science.

[43] Ohler A, Dubois A (2006). Phylogenetic relationships and generic taxonomy of the tribe Paini (Amphibia, Anura, Ranidae, Dicroglossinae), with diagnoses of two new genera.. Zoosystema.

[44] Che J, Hu J, Zhou W, Murphy RW, Papenfuss TJ (2009). Phylogeny of the Asian spiny frog tribe Paini (Family Dicroglossidae) sensu Dubois.. Molecular Phylogenetics and Evolution.

[45] Che J, Zhou W, Hu J, Yan F, Papenfuss TJ (2010). Spiny frogs (Paini) illuminate the history of the Himalayan region and Southeast Asia.. Proceedings of the National Academy of Sciences.

[46] Dubois A (1992). Notes sur la classification des Ranidae (Amphibiens Anoures).. Bulletin Mensuel de la Société Linnéenne de Lyon.

[47] Fei L, Hu S, Ye C, Huang Y (2009). Fauna Sinica, Amphibia Vol. 3. Anura Ranidae.

[48] Frost D (2010). Amphibian Species of the World: An Online Reference. Version 5.4 (8 April, 2010).

[49] Myers N, Mittermeier RA, Mittermeier CG, da Fonseca GAB, Kent J (2000). Biodiversity hotspots for conservation priorities.. Nature.

[50] Bordoloi S, Borah M, Chakravorty P, Sinha B (2001). First record of the Ranid frog *Paa annandalii* (Boulenger 1920) fromnorth-eastern region (Arunachal Pradesh) of India with a note on itslarval stages.. Current Science.

[51] Dubois A, Khan M (1979). A new species of frog (genus *Rana*, subgenus *Paa*) from northern Pakistan (Amphibia, Anura).. Journal of Herpetology.

[52] Dubois A, Matsui M (1983). A new species of frog (genus *Rana*, subgenus *Paa*) from western Nepal (Amphibia: Anura).. Copeia.

[53] Khan M, Tasnim R (1989). A new frog of the genus *Rana*, subgenus *Paa*, from southwestern Azad Kashmir.. Journal of Herpetology.

[54] Wang Z, Tang Z, Fang J (2007). Altitudinal patterns of seed plant richness in the Gaoligong Mountains, south-east Tibet, China.. Diversity and Distributions.

[55] Qian H (1998). Large-scale biogeographic patterns of vascular plant richness in North America: an analysis at the generic level.. Journal of Biogeography.

[56] Rohde K, Heap M, Heap D (1993). Rapoport's rule does not apply to marine teleosts and cannot explain latitudinal gradients in species richness.. American Naturalist.

[57] Jaccard P (1901). Distribution de la flore alpine dans le bassin des dranses et dans quelques regions voisines.. Bulletin Societe Vaudoise Des Science Naturelles.

[58] Brown WC, Alcala AC (1961). Populations of amphibians and reptiles in the submontane and montane forest of Cuernos Negros, Philippine Islands.. Ecology.

[59] Fauth JE, Crother BI, Slowinski JB (1989). Elevational patterns of species richness, evenness, and abundance of the Costa Rican leaf-litter herpetofauna.. Biotropica.

[60] Nathan R, Werner YL (1999). Reptiles and breeding birds on Mt. Hermon: patterns of altitudinal distribution and species richness.. Israel Journal of Zoology.

[61] Fu C, Hua X, Li J, Chang Z, Pu Z (2006). Elevational patterns of frog species richness and endemic richness in the Hengduan Mountains, China: geometric constraints, area and climate effects.. Ecography.

[62] Fischer J, Lindenmayer DB (2005). The sensitivity of lizards to elevation: a case study from south-eastern Australia.. Diversity and Distributions.

[63] Wiens JJ, Parra-Olea G, Garcia-Paris M, Wake DB (2007). Phylogenetic history underlies elevational biodiversity patterns in tropical salamanders.. Proceedings of the Royal Society B-Biological Sciences.

[64] Vetaas OR, Grytnes JA (2002). Distribution of vascular plant species richness and endemic richness along the Himalayan elevation gradient in Nepal.. Global Ecology and Biogeography.

[65] Oommen MA, Shanker K (2005). Elevational species richness patterns emerge from multiple local mechanisms in Himalayan woody plants.. Ecology.

[66] Fu C, Wang J, Pu Z, Zhang S, Chen H (2007). Elevational gradients of diversity for lizards and snakes in the Hengduan Mountains, China.. Biodiversity and Conservation.

[67] Li J, Song Y, Zeng Z (2003). Elevational gradients of small mammal diversity on the northern slopes of Mt. Qilian, China.. Global Ecology and Biogeography.

[68] Kessler M (2002). The elevational gradient of Andean plant endemism: varying influences of taxon-specific traits and topography at different taxonomic levels.. Journal of Biogeography.

[69] Brown JH (2001). Mammals on mountainsides: elevational patterns of diversity.. Global Ecology and Biogeography.

[70] Harrison T, Copeland P, Kidd W, Yin A (1992). Raising Tibet.. Science.

[71] Sanders NJ, Moss J, Wagner D (2003). Patterns of ant species richness along elevational gradients in an arid ecosystem.. Global Ecology and Biogeography.

[72] Webb TJ, Gaston KJ (2003). On the heritability of geographic range sizes.. American Naturalist.

[73] Grau O, Grytnes J-A, Birks HJB (2007). A comparison of altitudinal species richness patterns of bryophytes with other plant groups in Nepal, Central Himalaya.. Journal of Biogeography.

[74] Colwell RK, Hurtt GC (1994). Nonbiological gradients in species richness and a spurious Rapoport effect.. American Naturalist.

[75] Rohde K (1996). Rapoport's rule is a local phenomenon and cannot explain latitudinal gradients in species diversity.. Biodiversity Letters.

[76] Stotz D (1996). Neotropical Birds: Ecology and Conservation.

[77] Ruggiero A, Lawton JH (1998). Are there latitudinal and altidudinal Rapoport effects in the geographic ranges of Andean passerine birds?. Biological Journal of the Linnean Society.

[78] Kallimanis AS, Mazaris AD, Tzanopoulos J, Halley JM, Pantis JD (2008). How does habitat diversity affect the species–area relationship?. Global Ecology and Biogeography.

[79] Li J, Fang X (1999). Uplift of the Tibetan Plateau and environmental changes.. Chinese Science Bulletin.

[80] Meiri S, Dayan T (2003). On the validity of Bergmann's rule.. Journal of Biogeography.

[81] Blackburn TM, Ruggiero A (2001). Latitude, elevation and body mass variation in Andean passerine birds.. Global Ecology and Biogeography.

[82] Brehm G, Fiedler K (2004). Bergmann's rule does not apply to geometrid moths along an elevational gradient in an Andean montane rain forest.. Global Ecology and Biogeography.

[83] Hausdorf B (2003). Latitudinal and altitudinal body size variation among north-west European land snail species.. Global Ecology and Biogeography.

[84] Measey GJ, Van Dongen S (2006). Bergmann's rule and the terrestrial caecilian *Schistometopum thomense* (Amphibia: Gymnophiona: Caeciiiidae).. Evolutionary Ecology Research.

[85] Xie F, Lau M, Stuart S, Chanson J, Cox N (2007). Conservation needs of amphibians in China: a review.. Science in China Series C: Life Sciences.

[86] Parmesan C, Ryrholm N, Stefanescu C, Hill JK, Thomas CD (1999). Poleward shifts in geographical ranges of butterfly species associated with regional warming.. Nature.

[87] Hickling R, Roy DB, Hill JK, Thomas CD (2005). A northward shift of range margins in British Odonata.. Global Change Biology.

[88] Araújo MB, Thuiller W, Pearson RG (2006). Climate warming and the decline of amphibians and reptiles in Europe.. Journal of Biogeography.

[89] Hu J, Hu H, Jiang Z (2010). The impacts of climate change on the potential wintering distribution of an migratory bird.. Oecologia.

[90] Thomas CD, Cameron A, Green RE, Bakkenes M, Beaumont LJ (2004). Extinction risk from climate change.. Nature.

